# Emergency department charges may be associated with mortality in patients with severe sepsis and septic shock: a cohort study

**DOI:** 10.1186/s12873-018-0212-3

**Published:** 2018-12-29

**Authors:** Nicholas M. Mohr, Ryan Dick-Perez, Azeemuddin Ahmed, Karisa K. Harland, Dan Shane, Daniel Miller, Christine Miyake, Levi Kannedy, Brian M. Fuller, James C. Torner

**Affiliations:** 1 Department of Emergency Medicine, University of Iowa College of Medicine, 200 Hawkins Drive, 1008 RCP, Iowa City, IA 52246 USA; 2 Division of Critical Care, Department of Anesthesia, University of Iowa Carver College of Medicine, 200 Hawkins Dr. 6546 JCP, Iowa City, IA 52246 USA; 3 Department of Health Management and Policy, University of Iowa College of Public Health, N244 CPHB, Iowa City, IA 52246 USA; 4 TeamHealth, St. Rose Dominican Hospital-Siena Campus, Southern Hills Hospital and Medical Center, 2380 W. Horizon Ridge Parkway, Ste 110, Henderson, NV 89052 USA; 5 Division of Critical Care, Department of Anesthesiology, Division of Emergency Medicine, Washington University School of Medicine, One Brookings Drive, St. Louis, MO 63130 USA; 6 Department of Epidemiology, University of Iowa College of Public Health, S441A CPHB, Iowa City, IA 52246 USA

**Keywords:** Sepsis, Health services research, Costs and cost analysis, Risk adjustment, Emergency service, hospital, Critical illness

## Abstract

**Background:**

Sepsis severity of illness is challenging to measure using claims, which makes sepsis difficult to study using administrative data. We hypothesized that emergency department (ED) charges may be associated with hospital mortality, and could be a surrogate marker of severity of illness for research purposes. The objective of this study was to measure concordance between ED charges and mortality in admitted patients with severe sepsis or septic shock.

**Methods:**

Cohort study of all adult patients presenting to a 60,000-visit Midwestern academic ED with severe sepsis or septic shock (by ICD-9 codes) between July 1, 2008 and June 30, 2010. Data on demographics, admission APACHE-II score, and disposition was extracted from the medical record, and comorbidities were identified from diagnosis codes using the Elixhauser methodology. Summary statistics were reported and bivariate concordance was tested using Pearson correlation. Logistic regression models for 28-day mortality were developed to measure the independent association with mortality.

**Results:**

We included a total of 294 patients in the analysis. We found that ED charges were inversely related to mortality (adjusted OR 0.829 per $1000 increase in total ED charges, 95%CI 0.702–0.980). ED charges were also independently associated with 28-day hospital-free and ICU-free days (0.74 days increase per $1000 additional ED charges, 95%CI 0.06–1.41 and 0.81 days increase per $1000 additional ED charges, 95%CI 0.05–1.56, respectively). ED charges were also associated with APACHE-II score ($34 total ED charges per point increase in APACHE-II score, 95%CI $6–62).

**Conclusions:**

ED charges in administrative data sets are associated with in-hospital mortality and health care utilization, likely related to both illness severity and intensity of early sepsis resuscitation. ED charges may have a role in risk adjustment models using administrative data for acute care research.

## Background

Administrative data sets are an increasingly important tool for conducting population-based and health services research as they contain detailed information about medical care and outcomes [1]. Administrative data sets allow for rapid identification of a large cohort of patients, and they facilitate study across medical centers and health systems [2]. While health services studies using administrative data in sepsis and critical care exist [3, 4], it has been challenging to perform rigorous analyses because no robust physiologic measure of illness severity is captured in hospital claims [5]. Therefore there remains a need for a validated method to adjust for severity of illness based on administrative data.

Sepsis is responsible for 17% of US in-hospital deaths, and nearly one-half of sepsis admissions are treated in the emergency department (ED) [6, 7]. Age, medical comorbidities, admission diagnosis, and surgical status impact morbidity and mortality significantly, so risk adjustment models are critical to compare outcomes across clinical sepsis studies [8]. Illness severity scores, such as the Acute Physiology and Chronic Health Evaluation, 2nd edition (APACHE-II) score [9], the Simplified Acute Physiology Score (SAPS), and the Sequential Organ Failure Assessment (SOFA) score are valid scoring systems for critically ill patients [10–12], but the parameters used to calculate these scores are not captured in claims.

The objective of this study is to measure the concordance between ED charges and mortality in patients admitted with severe sepsis or septic shock, with a secondary objective of measuring the association between ED charges and APACHE-II score. We hypothesize that intensity of care is closely related to severity of illness, and that level of intensity could be approximated by ED charges. If validated, ED charges could be a useful covariate for claims-based health services analyses of acute care conditions like sepsis.

## Methods

### Study design

This study was a cohort study of all adult (age ≥ 18 years) patients presenting to a Midwestern academic 60,000-visit ED with severe sepsis or septic shock between July 1, 2008 and June 30, 2010. Severe sepsis and septic shock were defined by International Classification of Diseases, 9th Edition (ICD-9) hospital discharge diagnosis codes (995.92 or 785.52), evidence of infection during the index ED visit, and admission to the intensive care unit (ICU) during the hospital stay. Patients who had surgery performed were excluded from the data set. The study was approved by the local institutional review board (University of Iowa IRB-001, reference number 201211702) under waiver of informed consent.

### Study protocol

After charts were identified by ICD-9 criteria, a study investigator (CM) manually reviewed each medical record to confirm that the study subjects had infection in the ED. Then a research assistant, blinded to the study hypothesis, reviewed each medical record to abstract data using a standardized case report form. We collected data on demographics, source of infection, APACHE-II score at hospital admission, and final disposition. Two data sets were used in the study, clinical data to calculate the APACHE-II scores and administrative data set to extract ED charges and hospital discharge diagnosis codes.

Professional fees and hospital facility charges were maintained as separate variables. Comorbidities were defined from billing discharge diagnosis codes using the Elixhauser methodology, which defines a set of 30 conditions associated with clinical outcomes and health care utilization [13]. For calculation of APACHE-II scores, we used laboratory and vital sign values from the medical record, and we assumed laboratory values that were not measured were normal. We recorded hospital mortality and 28-day hospital-free and ICU-free days, which we defined as the number of days in the first 28 days after admission that a patient spent alive outside the hospital and the ICU, respectively. Using this measurement for length-of-stay is frequently done in studies of the critically ill to accurately account for censored data among non-survivors.

### Outcomes

The primary outcome of the study was concordance between ED charges and mortality. Secondary outcomes included the association between ED charges, APACHE-II score, and Elixhauser comorbidities with hospital survival and hospital length-of-stay (measured as 28-day hospital-free and ICU-free days).

### Analysis

We performed summary statistics using the Student’s t-test, the Wilcoxon rank-sum test, and the chi-squared test, as appropriate. We used Pearson correlation coefficient to describe bivariate concordance between ED charges and APACHE-II score. Each analysis was conducted with total ED charges, ED professional fees, and ED facility charges separately, and the charge with the strongest association with APACHE-II score was used in subsequent charge-based analyses.

Next, a logistic regression model was developed using the primary outcome of hospital mortality. Three separate models were constructed and compared using the Bayesian Information Criterion (BIC): one had APACHE-II score as a single predictor, the second had ED charges as a single predictor, and the third had the Elixhauser comorbidity variables reduced to its most parsimonious model. A subsequent logistic regression model was developed that included both APACHE-II score and ED charges, hoping to understand whether ED charges provided additional predictive power beyond that available with APACHE-II score alone.

Each continuous predictor was initially modeled as a categorical variable by dividing it into quintiles to test the assumption that the predictors were linearly associated with mortality. Once this assumption was confirmed, the final models used the original continuous variable as the predictor, if appropriate. These models were compared using the area under the curve (AUC) of the receiver operator characteristics analysis to compare the relative explanatory power of each index in predicting hospital survival.

For the secondary analysis, univariate linear regression models were constructed to measure the association between APACHE-II score and ED charges (separately) with 28-day hospital-free days and with 28-day ICU-free days.

We completed all analysis using Stata v. 13.1 (StataCorp, College Station, TX), and all results are reported using the Strengthening the Reporting of Observational Studies in Epidemiology (STROBE) guidelines [14].

### Availability of data and materials

Because the data used for this analysis included identifiable protected health information, the original data set is not available for release.

## Results

During the 2-year study period, 294 patients were included in the analysis, and 83 (28%) died. Table 1 shows the demographics, ED interventions, comorbidities and baseline vitals. The median APACHE-II score was 17 (rang 2 to 47), and the median ED charge was $3640 (IQR $2861 - $5234).

**Table 1:** Demographics and ED care for patients in study cohort. Baseline characteristics of the study population

Factor	All Patients (*n* = 294)	Survivors (*n* = 211)	Non-survivors (*n* = 83)	Difference (95%CI)
Male, n (%)	163 (55)	121 (57)	42 (51)	6.7 (− 6.0–19.4)
Age, mean (SD)	58.0 (16.0)	57.2 (16.3)	60.0 (15.1)	−2.6 (− 1.4–6.7)
ICU Admission, n (%)	220 (75)	161 (76)	59 (71)	5.2 (−5.9–16.3)
Fluids Administered over 24 h, liters (mean, SD)	4161 (2881)	4454 (2940)	3420 (2596)	1034 (307–1761)
Lactate, mmol/L (mean, SD)	3.2 (2.8)	3.0 (2.3)	3.9 (3.7)	−0.9 (− 1.6–0.2)
Triage Vital Signs				
Triage systolic blood pressure, mmHg (mean, SD)	108 (65)	109 (74)	106 (31)	3 (−13–19)
Triage heart rate, bpm (mean, SD)	107 (26)	109 (24)	103 (31)	6 (−1–12)
White blood cell count, cells/mL (mean, SD)	16.2 (10.2)	15.9 (10.1)	17.0 (10.3)	−1.1 (−3.7–1.5)
ED Charges, Total, dollars (median, IQR)	$3640 (2861 – 5234)	$3840 (2947 – 5661)	$3380 (2793 – 4356)	334 (71–653)
Hospital Facility Charges (median, IQR)	$2776 (2232 – 3706)	$2863 (2288 – 3772)	$2574 (2049 – 3264)	264 (63–482)
Professional Fees (median, IQR)	$760 (660–1496)	$1050 (681–1950)	$681 (660–1050)	19 (0–21)
Comorbidities				
Peripheral vascular disease, n (%)	17 (6)	10 (5)	7 (8)	−3.7 (− 9.7–2.3)
Paralysis, n (%)	17 (6)	14 (7)	3 (4)	3.0 (− 2.9–9.0)
Neurologic disorders, n (%)	35 (12)	27 (13)	8 (10)	3.2 (− 5.1–11.4)
COPD, n (%)	47 (16)	32 (15)	15 (18)	−2.9 (− 12.3–6.4)
DM with complications, n (%)	23 (8)	20 (9)	3 (4)	5.9 (−1.0–12.7)
Hypothyroidism, n (%)	23 (8)	17 (8)	6 (7)	0.8 (−6.0–7.7)
Renal failure, n (%)	38 (13)	29 (14)	9 (11)	2.9 (−5.7–11.5)
Liver disease, n (%)	42 (14)	23 (11)	19 (23)	−12.0 (−20.8 - -3.1)
Metastatic cancer, n (%)	18 (6)	11 (5)	7 (8)	−3.2 (−9.3–2.9)
Solid tumor without metastasis, n (%)	16 (5)	8 (4)	8 (10)	−5.8 (−11.6 - -0.1)
Coagulopathy, n (%)	53 (18)	30 (14)	23 (28)	−13.4 (−23.2 - -3.7)
Fluid and electrolyte disorders, n (%)	231 (79)	153 (73)	78 (94)	−21.4 (−31.7 - -11.3)
Deficiency anemia, n (%)	34 (12)	30 (14)	4 (5)	9.4 (1.2–17.5)
Hypertension, n (%)	96 (32)	7 (3)	5 (6)	17.0 (5.1–28.8)
APACHE-II score (mean, SD)	16.9 (7.0)	16.2 (6.5)	18.6 (7.8)	−2.4 (−4.1 - -0.6)
28-day ICU-free days (median, IQR)	22 (0–26)	25 (22–26)	0 (0–0)	25 (24–25)
28-day hospital free days (median, IQR)	12 (0–21)	18 (10–22)	0 (0–0)	18 (17–20)

### Mortality

ED charges were significantly associated with mortality, with higher ED charges being associated with lower mortality (adjusted OR 0.829 per $1000 total ED charges, 95%CI 0.702–0.980).

### Severity of illness

**Fig. 1:**
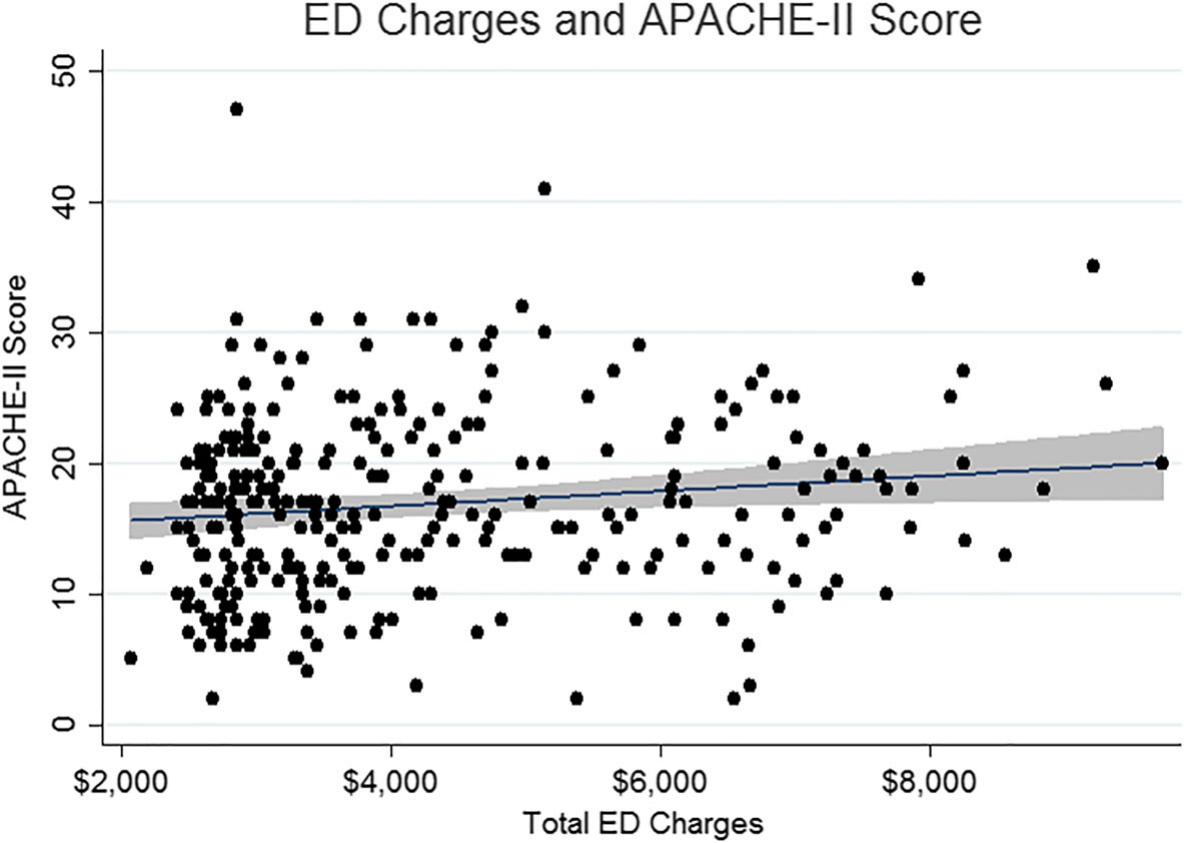
Scatter plot comparing ED charges with APACHE-II scores

Illness severity and ED charges were also associated (*p* = 0.016), with every point increase in APACHE-II score associated with a $34 (95% CI $6–62) increase in ED charges (Fig. 1). Of the total ED charges, both hospital charges ($20 per point increase in APACHE-II score, 95%CI $3–37) and professional fees ($14 per point increase in APACHE-II score, 95%CI $2–26) were correlated with APACHE-II score.

### Prediction of mortality

**Fig. 2:**
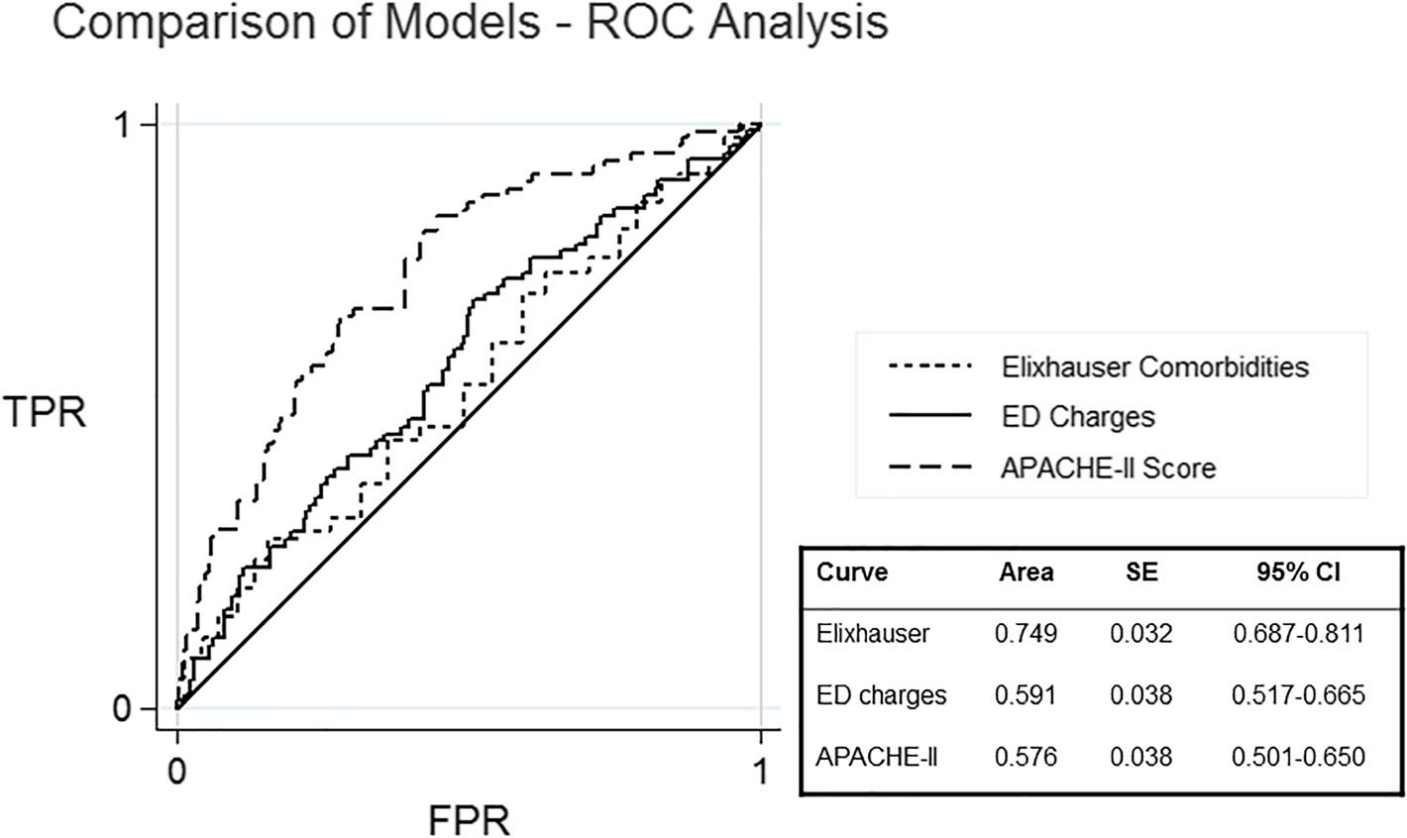
Receiver operator characteristics (ROC) curve comparing three variables on their ability to predict survival: Elixhauser comorbidities, ED charges, and APACHE-II scores

Elixhauser comorbidities, APACHE-II score, and ED charges all independently predict 28-day mortality (*p* < 0.001, *p* = 0.010, *p* = 0.028). Elixhauser comorbidities predicted mortality well (AUC = 0.729), while APACHE-II score and ED charges were significant but poor in their predictive value (AUC = 0.575 and AUC = 0.596, respectively) (Fig. 2). Both APACHE-II and ED charges were linearly related to mortality.

### Prediction of length-of-stay

ED charges were independently associated with 28-day hospital-free days (0.74 days increase in 28-day hospital-free days per $1000 additional ED charges, 95%CI 0.06–1.41) and 28-day ICU-free days (0.81 days increase in 28-day ICU-free days per $1000 additional ED charges, 95%CI 0.05–1.56). APACHE score was the stronger predictor of both hospital and ICU-free days (BIC 2188 vs. 2193 for hospital-free days, BIC 2254 vs. 2259 for ICU-free days).

## Discussion

The ability to adjust for severity of illness is important in comparing clinical outcomes such as mortality and length-of-stay in observational health services analyses, but this adjustment has been elusive for administrative analyses of sepsis studies. The APACHE-II score is a robust and well-validated tool that can be used to predict mortality, and it is commonly used when comparing health care outcomes for critically ill patients [10]. Physiologic and laboratory data are often unavailable in health services analyses that are conducted using administrative claims data, which limits severity adjustment in these studies [15]. This study identifies ED charges as a potential surrogate measure, suggesting that it can be used as a covariate in outcomes-based sepsis studies using administrative claims, even when more robust physiologic severity of illness measures are not available.

ED charges are usually viewed as an *outcome* related to care provided, but they are also a reflection of the amount and type of care *needed* during an ED stay. In the United States, ED charges are generated from professional fees and facility fees, and charges performed during the study period were performed on a fee-for-service basis. These fees are based on the time that health care providers spend caring for a patient, separately billable procedures that are performed (e.g., central venous line placement, endotracheal intubation), and the medical complexity documented. All severity of illness indices are surrogate markers for the probability of death assessed at admission [9]. These markers are designed to allow for risk adjustment to measure the probability of death attributable to only conditions recognized at the time of hospital admission, and ED charges were hypothesized to function similarly. Patients who are more seriously ill (hypotensive, requiring intubation, or needing more time and resource-intensive care) also receive higher hospital bills, and that relationship was validated in our model.

In addition to finding that ED charges are related to mortality, however, the decreased mortality was found with *higher* charges. This finding might indicate that care delivered in the ED (measured by charges) may also be *influencing* outcomes. Early aggressive ED sepsis care has been shown to decrease mortality [16], and the higher ED charges may be capturing the impact of aggressive appropriate resuscitation. This finding suggests that ED charges may be included in studies of non-ED interventions on sepsis outcomes, because controlling for ED charges may capture both the effect of severity of illness and treatment effects of ED care.

This study introduces ED charges as a predictor of clinical outcomes when physiologic severity of illness scores are not available retrospectively for research purposes. There are many areas of acute care research that may benefit from adjusting for ED charges as a marker of disease severity. Studies of diseases where acute disease severity strongly influences outcomes, such as sepsis, acute respiratory failure, stroke, and trauma may benefit from such an adjustment. Although these data only inform the use of ED charges in sepsis studies, other future studies should consider ED charges for its predictive value. In this way, ED charges can be treated as a nuisance variable – a variable that may be associated with a potentially confounding variable, but where the magnitude of effect is irrelevant in itself. There may also be other non-critical care diseases where this adjustment may also be useful, such as asthma or congestive heart failure.

## Limitations

This study has several limitations. First, we used retrospectively collected data from a single site. Although this may limit the external validity of our finding, it reflects accurately the way that this tool may be used in actual observational analyses. For our data collection we looked at ED charges, not actual cost to the patient. Costs are challenging to estimate, so they often are estimated based on the cost-to-charge ratio, and use of this ratio would be expected to yield similar precision. As with all claims-based studies, there is a bias towards identifying patients with more severe illness [17]. The high mortality in this cohort, however, supports that severity was likely reflected accurately. Finally, as a single-center study, we have no data on the concordance between hospitals, which is an important consideration for this method to be used on a large scale. Additionally, the application of ED cost to predict mortality may not be applicable outside of the United States where charges are more variable. Fortunately, studies that propose to use this method can validate the association between ED charges and clinical outcomes within the study, so using ED charges as a covariate in a multivariable regression model will allow one to assess its contribution to model fit in each application independently.

This study also included only patients admitted to an intensive care unit. These inclusion criteria were intended to limit the study population to those with greater severity of illness, but it may limit the generalizability of the findings.

## Conclusions

ED charges are a useful surrogate measure for severity of illness in sepsis studies based on administrative data. ED charges are inversely associated with mortality in severe sepsis and septic shock, and they predict hospital and ICU length-of-stay. ED charges may reflect both severity of illness and the contribution of ED care on clinical outcomes. Future work should focus on the applicability of comparing this covariate across hospital systems to adjust severity of illness in large administrative data sets, and to use it in other disease categories.

## Abbreviations

APACHE-II: Acute Physiology and Chronic Health Evaluation, 2nd edition
AUC: Area Under the Curve
BIC: Bayesian Information Criteria
ED: Emergency Department
ICD-9: International Classification of Diseases, 9th Edition
ICU: Intensive Care Unit
SAPS: Simplified Acute Physiology Score
SOFA: Sequential Organ Failure Assessment
STROBE: Strengthening the Reporting of Observational Studies in Epidemiology
